# A unilateral external fixator combined with bone transport and tibio-talar fusion for the treatment of severe postoperative infection of peri-ankle fractures: retrospective analysis of 32 cases

**DOI:** 10.1186/s13018-024-04586-2

**Published:** 2024-02-02

**Authors:** Qinghu Li, Xin Wang, Yonghui Wang, Fanxiao Liu, Baisheng Fu

**Affiliations:** 1 Department of Orthopedics, Shandong Provincial Hospital Affiliated to Shandong First Medical University, No.324, Road Jing Wu Wei Qi, Jinan, 250021 Shandong China; 2 Department of Anesthesiology, Shandong Provincial Hospital Affiliated to Shandong First Medical University, No.324, Road Jing Wu Wei Qi, Jinan, 250021 Shandong China

**Keywords:** Peri-ankle fractures, Infection, Unilateral external fixator, Bone transport, Tibio-talar fusion, Retrospective analysis

## Abstract

**Background:**

To investigate the clinical effects of a unilateral external fixator combined with bone transport and tibio-talar fusion in the treatment of severe postoperative infection of peri-ankle fractures.

**Methods:**

The clinical data of 32 patients (22 men and 10 women) with severe postoperative infection of peri-ankle fractures were retrospectively analyzed. Patients’ age ranged from 26 to 62 (mean, 42 ± 9.5) years old. The types of fractures were distal tibia fracture (25 cases), distal tibia and fibula fracture (5 cases), and talus fracture (2 cases). All patients underwent treatment with unilateral external fixation combined with bone transport and tibio-talar fusion. 6 patients with severe infection received two-stage treatment involving focal debridement and external fixation, osteotomy, and bone transport. The remaining 26 patients underwent debridement, external fixation, and osteotomy simultaneously. The length of bone transport, total fixation time of the external fixator, and postoperative complications were recorded for all patients. The efficacy of the treatment was assessed using the American Association of Foot and Ankle Society (AOFAS) ankle–hindfoot score.

**Results:**

Patients were followed up for 16–36 months, with an average follow-up time of 24 months. The length of tibia bone transport ranged from 5 to 15 cm, with a mean length of 8.5 cm. The external fixator was applied for 12–24 months, with an average duration of 16 months. One patient suffered from refracture at tibio-talar fusion site, and one patient had external fixation pin-tract infection. No complications, such as recurrent infections (especially the MRSA infection), poor mineralization, refracture, iatrogenic nerve damage or fusion failure, were found in the remaining patients. The preoperative AOFAS ankle–hindfoot function score was 40.0 ± 3.8 (range, 30–52) points, and it increased to 75.0 ± 3.0 (range, 67–78) points at the last follow-up.

**Conclusion:**

A unilateral external fixator combined with bone transport and tibio-talar fusion is an effective method for treating severe postoperative infection of peri-ankle fractures. This approach is capable of reconstructing large bone defects that remain after clearing the infected lesion. Additionally, it provides stability to the ankle, enhances ankle–hindfoot function, and improves the patient’s quality of life.

## Introduction

Fractures around the ankle joint are quite frequent among all bone fractures, representing approximately 10.2% of cases [1]. With the progress in transportation and construction industry, there has been an escalation in the occurrence of peri-ankle fractures caused by high-energy injuries. These incidents are becoming remarkably prevalent. These fractures are characterized by fracture comminution, severe soft tissue damage, and open injuries [2]. Common complications, such as fracture nonunion, infection, traumatic arthritis, and osteonecrosis, are easy to occur in the later stages [3, 4]. Managing postoperative infections can be quite challenging due to several factors [5], including inadequate blood supply, thin skin, and insufficient soft tissue coverage that make it more difficult to effectively treat these infections. Additionally, the presence of severe wound complications, as well as co-existing conditions, such as diabetes and peripheral arterial occlusive disease, further complicate the clinical treatment. The treatment of this condition poses numerous challenges, and the prognosis may be discouraging [6]. However, recent studies have shed light on the main objectives of the treatment approach. These include the complete eradication of the infection, achieving stable and painless joints, and restoring the patient’s ability to walk effectively [7].

In cases where patients experience severe infection and significant damage to the articular surface following surgery for peri-ankle fractures, clinical practice mainly involves primary debridement and drainage to control the infection. Additionally, secondary fusion or primary antibiotic bone cement implantation followed by secondary fusion is frequently employed treatment approaches. However, it is noteworthy that infections may reoccur, and joint fusion typically faces challenges with nonunion [8]. Prolonged irrigation can lead to the spread of infection throughout the ankle, compromising ankle function and affecting the entire ankle joint. Simple debridement may not fully address bone and joint cavity infections, increasing the risk of recurrent infection. Amputation, while capable of completely eliminating infected lesions, may pose challenges in terms of prosthesis functionality and significantly impact patients’ psychological well-being.

The key to successful treatment of peri-ankle infections is thorough clearance of both infected soft tissue and bone segments. For patients with short segmental bone defects, the initial step involves removing the infected bone, followed by a bone graft for ankle fusion in the second stage. However, in cases of long segmental bone defects, the sizable amount of bone graft required can present challenges, mainly resulting in incomplete grafting and impeding the bone’s healing process. This incomplete grafting may negatively impact bone healing. Alternative internal fixation methods have shown promise in enhancing joint fusion rates. However, for patients with infections, the compromised condition of the skin and soft tissue makes the use of internal fixation more susceptible to recurrent infections. Since the Ilizarov technique was proposed and named after a former Soviet doctor in 1951 [9], it has spread extensively all over the world. With advancements in Ilizarov technology, significant progress has been made in treating bone infections, bone defect, and bone nonunion or malunion through bone transport [10]. Many options and approaches are available for ankle fusion, and external fixation with compression has been used in many forms for ankle fusion, especially in the patients with infection and osteomyelitis [11]. Therefore, can bone transport also yield favorable outcomes for patients with infections around the ankle joint? Recently, the combination of a unilateral external fixator, bone transport, and tibio-talar fusion was utilized to successfully address severe postoperative infections in peri-ankle fractures, resulting in satisfactory results.

## Materials and methods

Between January 2015 and January 2020, a study conducted at the Provincial Hospital of Shandong First Medical University examined 32 cases of severe postoperative infection following peri-ankle fractures. The study group comprised 22 men and 10 women. The age of patients ranged from 26 to 62 (average, 42 ± 9.5) years old. The causes of injury were categorized as follows: 4 cases were due to bruising, 8 cases were caused by falling injuries, and 20 cases resulted from traffic accidents. The primary fractures were classified as closed in 9 cases and open in 23 cases. In terms of specific fractures, postoperative infection of the distal tibia fracture was identified in 25 cases, with 5 cases exhibiting both distal tibia and fibula fractures, and 2 cases involving talus fractures. The duration of the disease before admission ranged from 3 months to 10 years, with an average duration of 14.5 months. All patients were admitted to the hospital due to ankle joint infection. Among them, 25 patients exhibited poor skin healing and abnormal sinus exudation, while 8 patients had bone exposure. Upon admission, regular wound surface bacterial cultures were conducted as an initial diagnostic measure. And at least 3 parts in the deep tissue and the bone were taken for bacteria culture during the operation. Four patients had diabetes, but their blood sugar was well controlled during the treatment. The patients’ characteristics are listed in Table 1.

**Table 1:** Patients’ baseline data

Gender	
Male	22
Female	10
Age	26–62 years
Cause of injury	
Crash	4
High fall	8
Traffic injury	20
Initial injury	
Closed fracture	9
Open fracture	23
Diabetes	4, well controlled
Time before admission	3 months–10 years
Bacterial culture	
*Staphylococcus aureus*	18
MRSA	5
*Escherichia coli*	4
*Pseudomonas aeruginosa*	2
Mix infection	3
Length of transport	5–15 cm
Time of fix	12–24 months

**Fig. 1:**
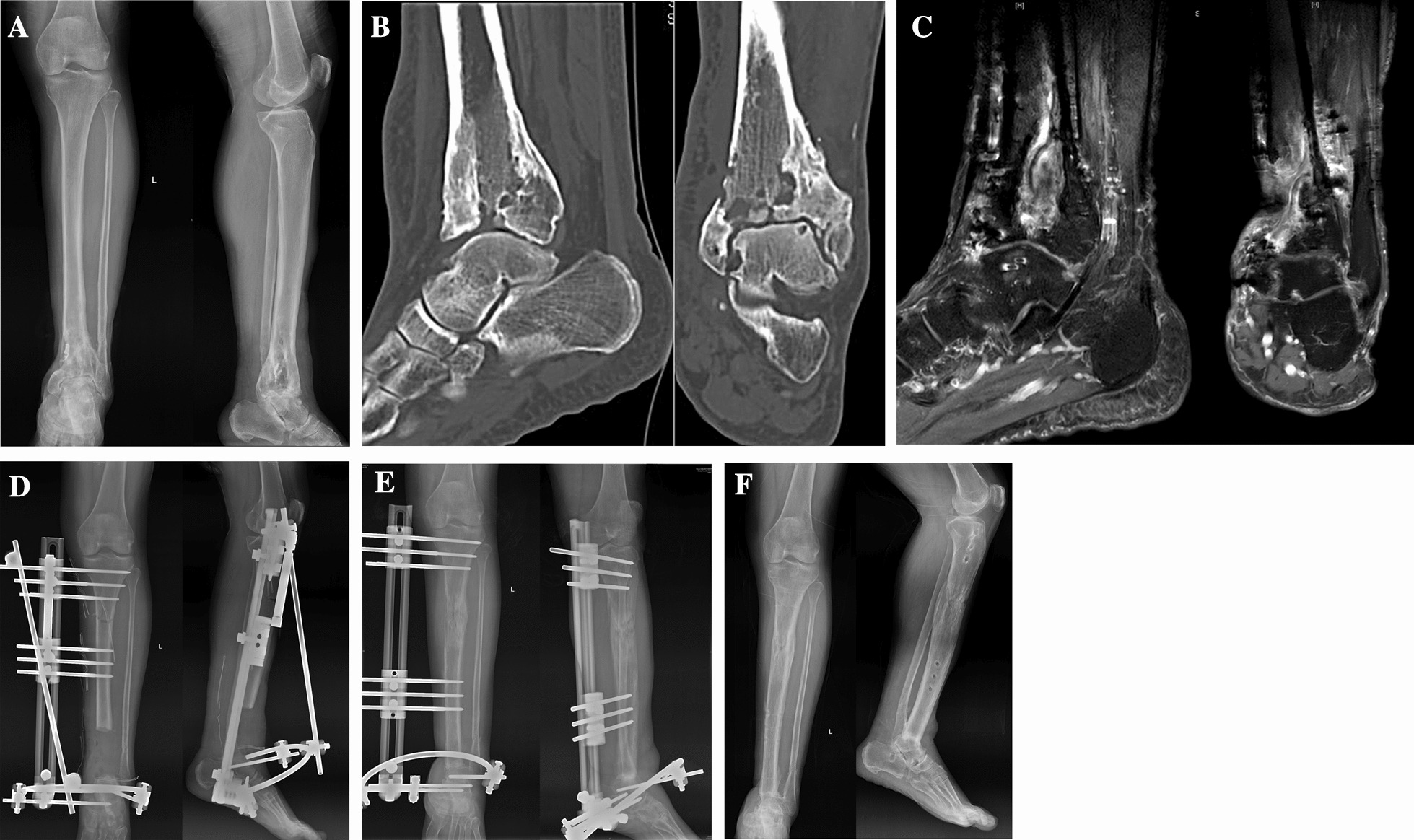
**A.** Postoperative left distal tibia fracture with intermittent abnormal exudation for 4 years. Upon transfer to our hospital, imaging revealed the presence of chronic osteomyelitis in the ankle joint area, accompanied by a wound healing disorder and joint destruction. **B.** CT image illustrating ankle joint destruction. **C.** MR image showing ankle joint destruction. **D.** Image was taken after complete removal of the infected lesions using unilateral external fixation and osteotomy. **E.** After 9 months of the surgery, noticeable mineralization of the new bone within the bone transport segment could be achieved. **F.** After 13 months of the surgery, successful tibio-talar fusion and excellent mineralization of the new bone could be achieved. Additionally, the external fixator was removed

X-ray, CT, and MRI were performed to confirm the presence of chronic osteomyelitis in the ankle joint area, accompanied by a wound healing disorder and joint destruction (Fig. 1A–C). The SPECT-CT or PET-CT all present good satisfactory accuracy for the diagnosis and delimitating the infection, but their costs should be further reduced to promote their wide application. All patients underwent comprehensive treatment, which involved complete removal of the infected lesions around the ankle joint, unilateral external fixation, bone transport, and tibio-talar fusion. In 6 patients, a staged approach was adopted, consisting of primary debridement and external fixation, followed by secondary osteotomy and bone transport to prevent the spread of infection. The remaining 26 patients simultaneously underwent debridement, external fixation, and osteotomy.

Following successful intraspinal anesthesia or general anesthesia, the infected tissue surrounding the ankle joint and the bone was carefully and completely removed. The wound was thoroughly rinsed with a large quantity of physiological saline. The distal end of the tibia was then amputated perpendicular to the anatomic axis, and the upper articular surface of the talus was chiseled off.

After disinfecting again and replacing the gloves and instruments, the surgery continued under fluoroscopy. First, determine the position of the fifth hole of the first clamp. The first hydroxyapatite (HA)-coated screw was inserted from the inside to the outside at this position by penetrating the contralateral cortex. Second, using this as a reference, the position of the middle clamp was adjusted to ensure sufficient sliding distance of the middle clamp after osteotomy. The second HA-coated screw was inserted, ensuring that the unilateral external fixation frame was parallel to the posterior edge of the tibia in the lateral position. Then, insert the rest screws. At the distal end of the unilateral external fixator, a T-clamp was installed. After placing the ankle joint in the neutral position, the talus was slightly moved inward and backward, and two screws were inserted vertically into the calcaneus through the T-clamp. To maintain stability, an additional fixation screw was inserted into the talus and secured with an accessory connected to the unilateral external fixator.

Minimally invasive osteotomy was performed at the proximal metaphyseal end of the tibia, approximately 15–20 mm away from the proximal clamp. A drill bit was initially used to drill holes, followed by an osteotome to cut off the tibia (Fig. 1D).

**Fig. 2:**
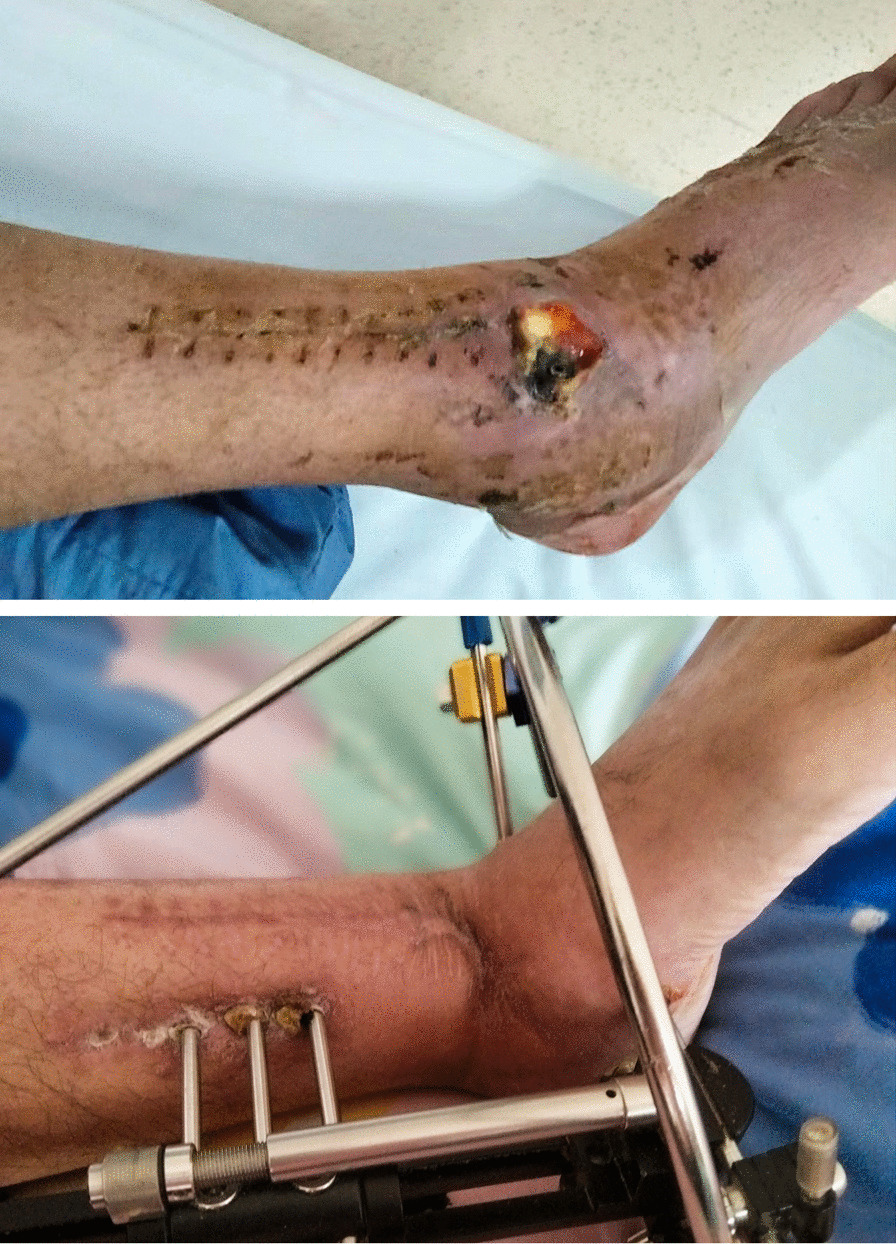
The skin and soft tissue was damaged and infected with purulent discharge before the treatment. And after the treatment, the skin and soft tissue was healed and no recurrence of infection

The wound can be closed one stage if it possible. If there is skin and soft tissue defect, dressing change can be selected during the bone transport. And the wound can heal during the bone transport (Fig. 2). The one-stage transfer of flap was not necessary. In cases where the skin was inset and there was insufficient contact between the bone ends after bone transport, it was necessary to perform surgical procedure at the union end. This procedure involved removing excessive soft tissue and ensuring a flat bone surface to promote effective tibio-talar fusion.

After the surgery, empirical broad-spectrum antibiotics were administrated, and sensitive antibiotics were then injected intravenously for 2 weeks according to the results of bacterial culture and drug sensitivity test. The drainage was pulled out within 72 h after the surgery.

The external fixator was adjusted for bone transport at 10 days after the surgery. The bone was incrementally lengthened by 1 mm per day, with adjustments made approximately 4 times on average. Following the surgery, it is important to encourage patient to engage in early mobilization and gradually increase the weight-bearing load based on the mineralization progress of the new bone. X-ray films were scheduled for reevaluation at 10 days after each adjustment to assess whether the direction and speed of bone transport would be corrected, and subsequently repeated every month until the tibia and talus would be contacted and reached an equal length **(**Fig. 1E**).** In the later stage, follow-up appointments should be scheduled every 2–3 months.

**Fig. 3:**
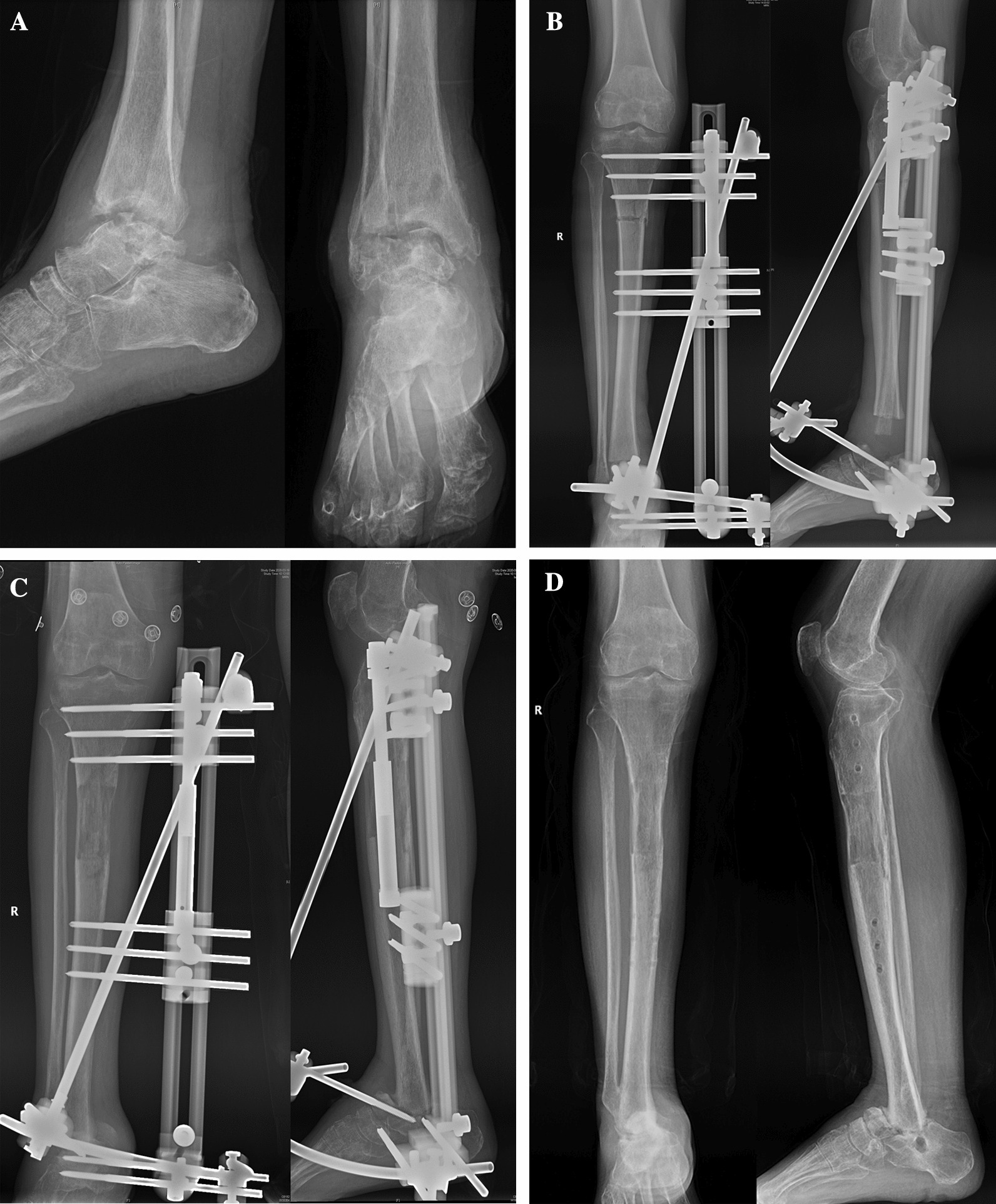
**A.** X-ray showed the infection of the talus and the infection-related bone destruction had spread the whole ankle joint. **B.** Postoperative X-ray showed the complete removal of the infected lesions using unilateral external fixation and osteotomy. **C.** The noticeable mineralization of the new bone within the bone transport segment could be achieved. **D.** Successful tibio-talar fusion and excellent mineralization of the new bone could be achieved. Additionally, the external fixator was removed

X-ray examination was performed to assess the mineralization of newly formed bone. Adequate bone mineralization was indicated by the presence of three distinct bone cortexes in four directions. Once confirmed, in conjunction with the healing of the tibio-talar fusion site, the fixation knob of the clamp could be removed to allow for dynamic movement. If there would be no apparent skeletal changes after one month of walking, the external fixator could be safely removed (Fig. 1F) and a case with talus fracture can be found in Fig. 3.

During the final follow-up, the American Association of Foot and Ankle Society (AOFAS) ankle–hindfoot score [12] was utilized to assess various parameters, including pain, walking ability, gait, range of motion, stability, and alignment. The score ranged as follows: 90–100 (excellent), 75–89 (good), 50–74 (fair), and below 50 (poor).

The statistical analysis was carried out using SPSS 20.0 software (IBM, Armonk, NY, USA). The data were expressed as mean ± standard deviation and analyzed using paired *t* test. *P* < 0.05 represented a statistically significant difference.

## Results

The culture results were summarized as follows: Staphylococcus aureus infection was detected in 18 cases, methicillin-resistant Staphylococcus aureus (MRSA) infection in 5 cases, Escherichia coli infection in 4 cases, Pseudomonas aeruginosa infection in 2 cases, and mixed bacterial infection in 3 cases.

All patients were followed up for 16–36 months, with an average follow-up time of 24 months. The length of tibial bone transport was 5–15 cm, with an average length of 8.5 cm. The fixation time of the external fixator was 12–24 months, with an average duration of 16 months. One patient suffered from refracture at tibio-talar fusion site after removal of external fixation and was recovered well with internal fixation. And one patient had external fixation pin-tract redness and swelling, and finally, the infection was controlled. And the remaining patients did not experience the complications, such as recurrent infections (especially the MRSA infection), poor mineralization, refracture, iatrogenic nerve damage, or fusion failure. The AOFAS ankle–hindfoot scores of the included cases are listed in Table 2. Prior to the surgery, the AOFAS ankle–hindfoot scores ranged from 30 to 52 (mean, 40.0 ± 3.8) points. However, at the last follow-up, the scores were significantly improved that ranged from 67 to 78 (mean, 75.0 ± 3.0), indicating a notable improvement (*P* < 0.05). Out of the total cases, 19 were classified as having good AOFAS ankle–hindfoot function, while 13 cases were deemed fair.

**Table 2:** The AOFAS ankle–hindfoot scores of the included cases

AOFAS ankle–hindfoot scores	Preoperative	Postoperative	P
Pain (max., 40 points)	20	40	
Activity limitations (max., 10 points)	4	10	
Walking distance (max., 5 points)	4	4	
Walking surface (max., 5 points)	3	3	
Gait abnormality (max., 8 points)	4	8	
Alignment (max., 10 points)	5	10	
Total score (max., 78 points)	40.0 ± 3.8	75.0 ± 3.0	< 0.05

## Discussion

The soft tissue around the ankle is generally weak, especially in cases of high-energy injuries resulting in peri-ankle fractures. These fractures are mainly accompanied by severe soft tissue injuries, and even open wounds. In clinical practice, it is necessary to perform open reduction and utilize multiple internal fixators to achieve proper alignment of the fractures and ensure stable fixation [13, 14]. However, this approach may result in remarkable skin and soft tissue damage, leading to complications, such as postoperative necrosis, infection, and ultimately bone infection and bone defects. When infections occur, they may exacerbate the challenges of treatment due to the inadequate and limited coverage of soft tissue. Infections can spread to the ankle joint, resulting in the destruction of the articular surface and invading surrounding tissues, including the talus. This may ultimately result in loss of joint function [15].

In cases of postoperative infection following an ankle joint fracture, the conventional approach involves comprehensive debridement to remove infected tissue, followed by flap transfer to cover the soft tissue defect. Fractures are treated using internal or external fixation methods. However, the traditional approach has limitations. It may not completely eliminate the infected bone due to concerns about preserving limb length, leading to residual infection, recurrent episodes, prolonged treatment duration, and unsatisfactory outcomes. But complete excision of the lesion may inevitably result in bone defect and ankle joint loss, necessitating secondary arthrodesis. Arthrodesis is typically employed as a salvage procedure to alleviate pain and maintain ankle stability [16, 17]. In our approach, we ensured thorough removal of the infected bone segment and addressed the challenges of remarkable bone defects through the utilization of Ilizarov technology with a unilateral external fixator. This technique allows for the retention of limb length while providing a compressive and elastic external fixator that facilitates fusion between the tibia and talus. Patients reported satisfactory ankle and hindfoot function, demonstrating positive therapeutic outcomes.

Thorough debridement serves as the foundation of treatment. Infected soft tissue, bone and cartilage should be excised thoroughly until fresh blood would ooze from the bone end. Inadequate debridement is a frequent underlying factor in therapy failure in patients with osteomyelitis [18]

The installation of an external fixator should be performed following debridement to maintain an aseptic operating environment and minimize the risk of recurrent infection. Accurate and effective placement of external fixators is crucial for successful outcomes. In the initial stage, it is essential to ensure that the path of the unilateral external fixator runs parallel to the tibial anatomical axis in both the anteroposterior and lateral positions. The initial HA-coated screw is of utmost importance, and its positioning should be adjusted promptly under fluoroscopy to ensure that it is perpendicular to the tibial anatomical axis. In the lateral position, it is crucial to consider the curvature of the tibia and the enlargement of the bone ends. The reference for nail placement should be the extension of the posterior edge of the tibia shaft, following a straight line. It is necessary to consider the slight deviation of the central axis of the tibia toward the inner side of the talus axis. In order to promote proper weight-bearing and foot alignment, the talus should be repositioned inward intraoperatively. Simultaneously, it is essential to carefully move the talus back to ensure effective weight distribution and support [19].

Performing proximal tibia osteotomy after the installation of external fixation is recommended. To minimize the risk of infection, it is important to keep the osteotomy instrument separate from the debridement instrument prior to the procedure. In cases where there was a potential for infection spreading to the proximal end, a second stage osteotomy should be considered to prevent subsequent infection at the osteotomy site.

Patients who have skin and soft tissue defects may not require an immediate transfer flap for wound coverage. In cases with skin and soft tissue defect, an open wound can be managed through regular dressing changes. During bone transport, the skin and soft tissues are stretched and elongated, allowing most of the wounds to eventually be reduced and closed [20]. However, in some patients with limited skin and soft tissue availability following bone transport, a second-stage flap repair can be easily performed to address the deficiency.

Regular follow-up is necessary after the surgery. It is recommended to monthly review the X-ray film to monitor the progress of bone transport, lower limb axis, and osteogenic effect. If osteogenesis is unsatisfactory, it may be necessary to slow down the transportation speed accordingly. Once the bone ends have aligned, the external fixation can be further adjusted to apply appropriate compression and ensure full contact. The docking site may have many complications, such as soft tissue incarceration and the delayed union or nonunion, so the docking site management is important [21]. If there was the soft tissue incarceration and no sign of healing at 3 months after the reunion, surgical cleaning of the docking site may be required. In such cases, autologous iliac bone can be utilized to fill the reunion site and promote bone healing.

In the treatment of peri-ankle fracture infection, it is crucial to identify the specific pathogenic bacteria involved and select appropriate antibiotics that are sensitive to them. This can be accomplished through bacterial culture and drug sensitivity testing. The timely administration of sensitive antibiotics can effectively control the infection and minimize the development of bacterial resistance typically associated with broad-spectrum antibiotics. According to the available literature, the most commonly reported pathogenic bacteria in such cases include Staphylococcus aureus (approximately 30% of cases), mixed bacteria (around 27% of cases), as well as other pathogens (e.g., coagulase-negative Staphylococci and gram-negative bacilli) [22]. In order to enhance the detection rate of pathogenic bacteria, it is important to perform deep tissue biopsies during bacterial culture. This method is effective in accurately identifying and isolating bacteria from the affected tissues [23]

Extensive debridement of postoperative infection in peri-ankle fractures may lead to remarkable bone defects that require reconstruction. At present, there are several common clinical methods for addressing this issue, including autogenous bone graft, vascularized free fibula graft, and Ilizarov bone transport [24]. Autogenous bone graft typically involves extracting bone from the iliac region. This method possesses advantages, such as relatively simple surgical procedures and a shorter treatment period. Additionally, as the amount of available bone from the donor site is limited, it may not meet the requirements for treating large segment bone defects adequately. On the other hand, vascularized free fibula graft relies on microsurgical techniques and has a steep learning curve [25]. The Ilizarov bone transport technique is widely utilized. This technique relies on reliable fixation and gradual traction to achieve distraction osteogenesis successfully. It has shown effectiveness in treating bone infections with bone defects [26, 27].

A unilateral external fixator combined with bone transport and tibio-talar fusion may possess several advantages for treating postoperative infection of peri-ankle fractures: (1) Complete removal of infected lesions: Using this approach, the infected areas can be entirely eliminated, regardless of the size of the resulting bone defect; (2) Resolution of large segmental bone defect and unequal limb length: The Ilizarov technique, combined with bone transport, can effectively address both large segmental bone defect and differences in limb length [28]; (3) Improved local blood circulation and enhanced infection treatment: The process of bone transport during this treatment method can improve local blood circulation, leading to more effective treatment of infection in the affected area; (4) Simplicity and convenience of unilateral external fixation: This approach offers a simple and convenient method of fixation for patients. The skin incisions required are relatively minimal, and patients tend to have high compliance with the treatment [29]; (5) Flexibility for realignment: When the alignment of the bone is not optimal, a unilateral external fixator can be readily adjusted in a timely manner, allowing for a proper realignment [30]; (6) The utilization of a unilateral external fixator provides a satisfactory stability, and it is allowed for early load-bearing walking. Applying continuous pressure to the convergence end can be beneficial in enhancing the fusion rate [31, 32]; and (7) There is no need for secondary fixation at the docking site, which avoids the recurrence of infection and damage to skin and soft tissue again.

However, there are still some shortcomings that are summarized as follows: (1) The external fixation method requires a long period of time for proper healing, leading to discomfort for the patient; (2) There is a risk of pin-tract infections, necessitating regular care and timely treatment to manage infection and drainage; (3) This technique demands a solid understanding of physics and biomechanics, and it also requires extensive training due to a steep learning curve [33]; and (4) After tibio-talar fusion, the structural changes in the ankle joint may lead to dysfunction [34], ultimately affecting an individual’s ability to walk and carry out daily activities. Additionally, this can result in the increased pressure on the surrounding joints, potentially speeding up the degeneration of adjacent joints [35]. While the utilization of external fixators for bone transport is accompanied by certain disadvantages, numerous complications can be effectively managed through early detection and proactive measures [36]. This method can be used to treat serious postoperative infections of peri-ankle fractures.

Treating postoperative infections associated with peri-ankle fractures can be quite challenging. It typically involves lengthy treatment, being costly, and limited effectiveness, while carrying the risk of complications. Therefore, prevention is the optimal approach. In cases of open fractures, a recommended method for final fixation is the combination of an external fixator and limited internal fixation. It is important to prioritize closing the wound within a 72-h timeframe to minimize the risk of infection. If a bone defect is present, secondary repair should be taken into account. In the case of closed fractures where the skin remains intact, whereas noticeable swelling is observed, an external fixator can be employed in the initial stage to preserve the length and alignment of the affected limb. Subsequently, during the second stage, when the soft tissue condition has significantly improved, limited open reduction and fixation of the fracture can be performed.

For severe postoperative infection of peri-ankle fractures, effective treatment options include extensive debridement, unilateral external fixation, bone transport, and tibio-talar fusion. This method aims to maximize the recovery of ankle–hindfoot function, stabilize the ankle joint, and improve the patient’s quality of life. However, this is a retrospective clinical analysis with a limited sample size and a short follow-up time. Additional large-scale prospective studies with long-term follow-up are required to confirm the findings.

## Data Availability

The datasets used and analyzed during the current study are available from the corresponding author on reasonable request.
